# The world of wound care loses a true legend: Dr. Barbara Braden

**DOI:** 10.1111/iwj.14321

**Published:** 2023-07-17

**Authors:** Keith Harding, Douglas Queen

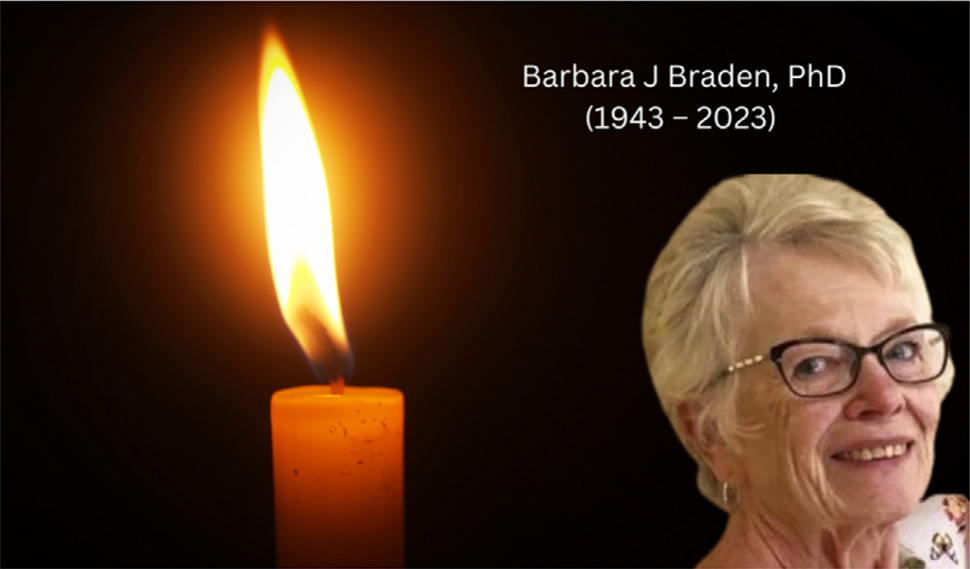

The IWJ family were saddened to learn of the passing of a wound care legend Dr. Barbara Braden after a 2‐year battle with cancer. While many of us may not have personally met Barbara, her name is known universally.

Barbara J. Braden, PhD, FAAN, was the Dean of University College at Creighton University in Omaha, Nebraska. She received her bachelor's degree from Creighton University in 1973, her master's from the University of California at San Francisco in 1975 and her doctoral degree from the University of Texas at Austin in 1988. She is best known for her work in the development of the Braden Scale for Predicting Pressure Sore Risk, which has been translated into many languages and is used on all continents. Dr. Braden is a Fellow of the American Academy of Nursing, and a past member of the NPUAP board of directors.

In her own words in an interview with Wounds Canada in 2007, she explained her surprising journey. ‘I authored the tool out of my expertise in clinical nursing but working with Dr. Nancy Bergstrom to test it convinced me to get my PhD. It has propelled me from being known locally and regionally to being known nationally and internationally. It has resulted in opportunities to speak to multidisciplinary audiences around the world. Because of it, I have received awards for achievement from the Creighton University, University of California at San Francisco and the University of Texas at Austin. It took me from faculty to administration and directly resulted in my appointment as Graduate Dean at Creighton University. Many people read this and interpret it as meaning I was in charge of graduate programs in nursing. But because of my international reputation for research in the field, the faculty of the university had sufficient respect for my expertise to make me Dean of the entire Graduate School, which included 21 master's programs and three doctoral programs across five different schools. In short, it turned a nice and moderately successful career into an unbelievably fabulous career!’^1^

It may have been surprising to her but not to those who knew her. Being the namesake of the most widely used pressure injury risk assessment globally changed her career. Dr. Braden had a major impact in nursing research and alongside her colleague Dr. Nancy Bergstrom changed pressure ulcer care forever. She was a leader in supporting nursing research and a strong advocate for nursing science and evidenced based care. Her passing while sad, reminds us all of the extensive influence of Barbara Braden in the wound‐care area and she will leave a legacy in this area for other clinicians and researcher to benefit from. Her legacy will live on through the continued work of many others, using the Braden Scale.

We all know she made the world a better place. Let us face it we all know that nurses make the world a better place. Plain and simple, they are the engine of healthcare. A legacy like the Braden Scale is testament to this impact being an excellent reminder of the difference nurses can make.

Dr. Braden, we thank you for all you contributed to our world of wound care. We thank you for making a difference in the lives of clinicians caring for those with wounds. But most of all we thank you for your impact in reducing the pain and suffering of those with or having the potential for pressure injuries.

The world has Superman and Superwoman but has sadly lost its Supernurse! Rest In Peace, and make sure those in heaven know how to use the Braden Scale effectively!
